# Growth hormone stimulates lipolysis in mice but not in adipose tissue or adipocyte culture

**DOI:** 10.3389/fendo.2022.1028191

**Published:** 2023-01-04

**Authors:** Lidan Zhao, Honglin Jiang

**Affiliations:** School of Animal Sciences, Virginia Tech, Blacksburg, VA, United States

**Keywords:** adipocytes, adipose tissue, growth hormone, lipolysis, adrenergic

## Abstract

The inhibitory effect of growth hormone (GH) on adipose tissue growth and the stimulatory effect of GH on lipolysis are well known, but the mechanisms underlying these effects are not completely understood. In this study, we revisited the effects of GH on adipose tissue growth and lipolysis in the *lit/lit* mouse model. The *lit/lit* mice are GH deficient because of a mutation in the GH releasing hormone receptor gene. We found that the *lit/lit* mice had more subcutaneous fat and larger adipocytes than their heterozygous *lit/+* littermates and that these differences were partially reversed by 4-week GH injection. We also found that GH injection to the *lit/lit* mice caused the mature adipose tissue and adipocytes to reduce in size. These results demonstrate that GH inhibits adipose tissue growth at least in part by stimulating lipolysis. To determine the mechanism by which GH stimulates lipolysis, we cultured adipose tissue explants and adipocytes derived from *lit/lit* mice with GH and/or isoproterenol, an agonist of the beta-adrenergic receptors. These experiments showed that whereas isoproterenol, expectedly, stimulated potent lipolysis, GH, surprisingly, had no effect on basal lipolysis or isoproterenol-induced lipolysis in adipose tissue explants or adipocytes. We also found that both isoproterenol-induced lipolysis and phosphorylation of hormone-sensitive lipase were not different between *lit/lit* and *lit/+* mice. Taken together, these results support the conclusion that GH has lipolytic effect in mice but argue against the notion that GH stimulates lipolysis by directly acting on adipocytes or by enhancing β-adrenergic receptors-mediated lipolysis.

## Introduction

Growth hormone (GH), a polypeptide hormone produced by the anterior pituitary, is a major regulator of growth and metabolism. Many studies have demonstrated an inhibitory effect of GH on adipose tissue growth (1–3). In humans and mice, GH deficiency increases fat deposition whereas GH replacement treatment reverses this increase (3–5). It is believed that GH decreases body fat mass by both reducing lipogenesis and increasing lipolysis (6–8).

The lipolytic effect of GH has been observed in a variety of animals, including mice (9), rats (10), cattle (11), and humans (12, 13). However, studies of the underlying mechanism have generated seemingly conflicting results. In adipocytes derived from mouse 3T3-F442A preadipocytes and epididymal fat explants from rats, GH stimulated the release of glycerol, an indication of lipolysis (14, 15). These results suggest that GH directly acts on adipocytes to stimulate lipolysis. However, GH had no lipolytic effect on adipocytes derived from 3T3-L1 cells (16) or human adipose tissue (17). These results do not support a direct lipolytic effect of GH on adipocytes or adipose tissue. In human omental adipose tissue explants and adipocytes isolated from rat parametrial adipose tissue, the lipolytic effect of GH was undetectable in the absence of glucocorticoids (18, 19). These results suggest that the lipolytic effect of GH is dependent on other lipolytic hormones.

Epinephrine and norepinephrine are strong lipolytic hormones, and they cause fat breakdown by binding to the β-adrenergic receptors (20). Growth hormone administration significantly improved the lipolytic response of isolated adipocytes to epinephrine (21). Similar enhancing effect of GH was also observed in rat adipocytes (22). These results have led to the notion that GH stimulates lipolysis by enhancing the direct lipolytic effect of epinephrine or norepinephrine on adipocytes (17, 22). Because the mechanism by which GH increases lipolysis is not clear, we determined the effect of GH on lipolysis and explored the underlying mechanism in a GH-deficient mouse model.

## Materials and methods

### Animal experiments

Breeding pairs of C57BL/6J-*Ghrhr^lit^* mice were purchased from The Jackson Laboratory (Bar Harbor, ME, USA). The *lit* mutation is a mutation in the growth hormone releasing hormone receptor (*Ghrhr*) gene, and homozygous *lit/lit* mice are GH deficient (23). Male *lit/lit* mice and male heterozygous littermates (*lit/+*) were used in this study. *Lit/+* littermates were used as controls for *lit/lit* mice because wild-type littermates were not available and because *lit/+* mice did not differ from wild-type mice in body weight (24). Mice were housed on a 12 h light/dark cycle at 23°C with *ad libitum* access to food and water. Mice were euthanized by CO_2_ inhalation and cervical dislocation. Euthanasia was performed between 9:00 a.m. and 11:00 a.m. All animal procedures used in this study were approved by the Virginia Tech Institutional Animal Care and Use Committee.

Four mouse experiments were performed in this study. In the first mouse experiment, 9-week-old *lit/lit* and *lit/+* mice were injected subcutaneously with recombinant bovine GH (The National Hormone and Peptide Program, Torrance, CA, USA) at 2 μg/g body mass or with an equal volume of control (0.01 M NaHCO_3_, vehicle for GH), at approximately 9:00 a.m., once a day for 4 weeks. Bovine GH was used because it was easier to obtain than mouse or human GH and because it was effective in stimulating body growth and IGF-I gene expression in mice, based on a previous study (25). Mice were weighed weekly at 9:00 a.m. during this experiment. Mice were euthanized for isolation of inguinal subcutaneous fat pads at the end of the experiment. In the second mouse experiment, 17-week-old mice were injected subcutaneously with recombinant GH or control once a day for 4 weeks as described above, and euthanized for isolation of the inguinal fat pads at the last day of the experiment. The inguinal fat pads in this study referred to the subcutaneous fat pads between the lumbar spine and the groin. In the third mouse experiment, the inguinal subcutaneous fat pads were taken from 13-week-old mice for explant culture and for isolation of the stromal vascular fraction (SVF). In the fourth mouse experiment, 13-week-old mice were injected intraperitoneally with 10 μg/g body mass of isoproterenol (Sigma-Aldrich, St. Louis, MO, USA); 15 minutes after the injection, mice were euthanized for collection of blood and fat tissue. In all of these experiments, food was not removed from mice prior to drug administration or euthanasia.

### Histology

Adipose tissue samples were fixed in 10% formalin (Fisher Scientific, Pittsburg, PA, USA) for approximately 18 h, and then embedded in paraffin for sectioning. Tissue sections were stained with hematoxylin and eosin (Fisher Scientific). Micrographs were randomly taken from each section. The areas of adipocytes in ten randomly selected micrographs, each of which contained around 150 adipocytes, were measured for each mouse using the ImageJ software (NIH, Bethesda, Maryland, USA).

### Culture of adipose tissue explants

Freshly isolated inguinal subcutaneous fat pads were cut into pieces of 5 to 10 mg. Fat tissue pieces were incubated in Krebs Ringer bicarbonate buffer (pH 7.4) supplemented with 4% of bovine serum albumin (BSA), 1 mg/ml of glucose (Fisher Scientific), and 100 ng/ml of recombinant bovine GH or equal volume of PBS at 37°C in an atmosphere of 5% CO_2_ and 90% humidity. Medium samples were taken at 4 h and 24 h of culture. Medium samples were immediately centrifuged to remove any debris and stored at -20°C for glycerol assay.

### Isolation of the stromal vascular fraction from fat

Freshly collected inguinal subcutaneous fat pads were cut into small pieces and then digested with 1 mg/ml collagenase D (Roche, Indianapolis, IN, USA) in HEPES buffer (0.1 M HEPES, 0.12 M NaCl, 0.05 M KCl, 5 mM glucose, 1.5% BSA, and 1 mM CaCl_2_, pH 7.4) at 37°C with shaking at 115 rpm for 1 h. The digestion was subsequently filtered through a 240 µm (pore size) mesh filter followed by a 40 µm mesh filter. The filtrate was centrifuged at 400 ×g for 5 min, and the pellet, i.e., the SVF, was washed twice with the HEPES buffer and then resuspended in growth medium. The growth medium consisted of Dulbecco’s Modified Eagle’s Medium Nutrient Mixture F-12 Ham (DMEM/F12) (Mediatech, Manassas, VA, USA), 10% of fetal bovine serum (FBS) (Atlanta Biologicals, Lawrenceville, GA, USA), 2 mM of L-glutamine (Mediatech), and 1% of 100× antibiotics-antimycotics (ABAM) (Mediatech).

### Culture of the stromal vascular fraction cells

The SVF cells were expanded in growth medium for approximately 4 days. The SVF cells were induced to differentiate into adipocytes as described before (26). In brief, the SVF cells at nearly 100% confluency were cultured in DMEM/F12 medium supplemented with 5% FBS, 1% ABAM, 2 mM L-glutamine, 17 nM insulin, 0.1 µM dexamethasone, 250 µM 3-Isobutyl-1-methylxanthine (IBMX), and 60 µM indomethacin (MP Biomedical, Solon, OH), for 2 days. Insulin, dexamethasone, and IBMX were all purchased from Sigma-Aldrich. The SVF cells were then cultured in DMEM/F12 supplemented with 10% FBS, 2 mM L-glutamine, 1% ABAM, and 17 nM insulin for 2 days. Lastly, the SVF cells were cultured in DMEM/F12 supplemented with 10% FBS, 2 mM L-glutamine, and 1% ABAM, for 4 days. To determine the effect of GH on lipolysis, the SVF cells on the 8^th^ day of differentiation were treated with 100 ng/ml recombinant bovine GH or control in serum- and insulin-free medium for 4 h and 24 h, and medium samples were taken for glycerol assay.

### Glycerol and non-esterified fatty acid assays

Concentrations of non-esterified fatty acids (NEFA) in serum samples (7 μl) were measured as indicators of lipolysis in mice using the HR Series NEFA HR kit from FUJIFILM Wako Diagnostics (Richmond, VA, USA). Concentrations of NEFA in medium samples were undetectable using the HR Series NEFA HR kit; therefore, concentrations of glycerol were measured as indicators of lipolysis in cultured adipose explants and adipocytes. Glycerol concentrations in medium samples (10 μl) were measured using the Glycerol Colorimetric Assay Kit from Cayman Chemical (Ann Arbor, Michigan, USA). These assays were performed essentially according to the manufacturers’ instructions. Each sample was analyzed in duplicate. The detection limit of the glycerol assay was 2.5 mg/L or 0.027 mM, and the detection limit of the NEFA assay was around 1 mEq/L or 1 mM according to the manufacturers’ manuals.

### Western blot analysis

Adipose tissue (200-300 mg) was homogenized in 1 ml of radioimmunoprecipitation assay (RIPA) buffer (50 mM Tris-HCl, pH 8.0, 150 mM NaCl, 1.0% NP-40, 0.1% SDS, 0.5% sodium deoxycholate) supplemented with protease inhibitors and phosphatase inhibitors (Roche). The homogenate was centrifuged at 10,000 ×g for 20 min at 4°C. The clear phase was collected and centrifuged again at 13,000 ×g for 10 min. Protein concentrations were measured using a BCA protein assay kit (Thermo Scientific, Rockford, IL, USA). Equal amounts of protein samples were separated by 8% SDS-PAGE and transferred electrophoretically onto nitrocellulose membranes. The membranes were immunoblotted first with 1:1000 diluted antibody for phosphorylated hormone sensitive lipase (Phospho-HSL, Ser563) (Cell Signaling, Danvers, MA, USA) and then with 1:1000 diluted antibody for total HSL (Santa Cruz Biotechnology, Santa Cruz, CA, USA). Primary antibodies were detected by 1:1000 diluted horseradish peroxidase-coupled IgG (Santa Cruz Biotechnology) followed by Pierce ECL substrate (Thermo Scientific). Chemiluminescent signals were detected by exposing the membrane to a CL-Xposure film (Thermo Scientific, Rockford, IL, USA).

### Statistical analysis

ANOVA followed by Tukey’s test was used to compare multiple means. T-test was used to compare two means. These analyses were performed using the General Linear Model of JMP (SAS Institute Inc., Cary, NC, USA). All data are expressed as mean ± SEM (standard error of the mean).

## Results

### GH deficiency increased adipose tissue growth

**Figure 1:**
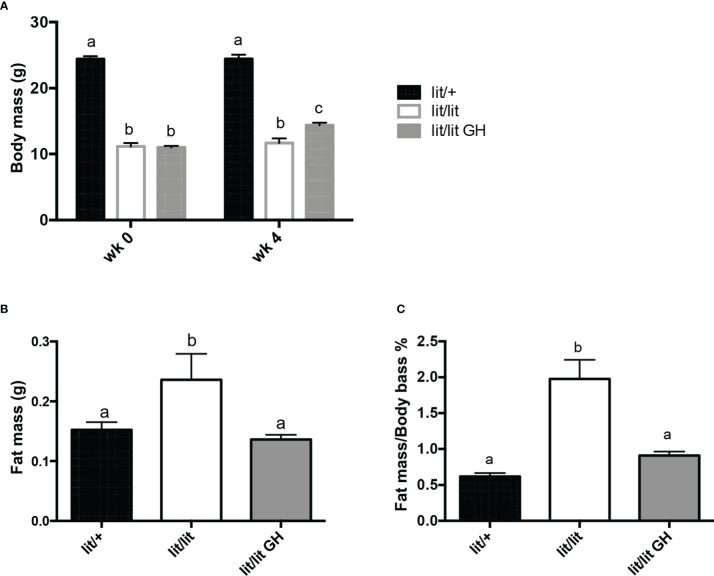
Effects of GH deficiency and GH injection on body mass and fat mass of mice. Three groups of 9-week-old male *lit/lit, lit/lit,* and *lit/+* mice from the same litters were injected with 2 μg/g body mass of recombinant bovine GH, vehicle control, and vehicle control, respectively, once a day for 4 weeks. Body mass and mass of inguinal subcutaneous fat pads were recorded at the beginning (wk 0) and end (wk 4) of the experiment. **(A)** Body mass. **(B)** Mass of inguinal subcutaneous fat pads. **(C)** Inguinal fat pad mass as percentage of body mass. Data are expressed as mean ± SEM (n = 5 mice). Means labeled with different letters are statistically different (*P* < 0.05).

To determine the effect of GH deficiency on adipose tissue mass, we compared body mass and inguinal fat pad mass among 13-week-old *lit/lit* mice, *lit/+* mice, and *lit/lit* mice that had been injected with GH for the last 4 weeks. Before GH injection (i.e., at 9 weeks of age), average body mass of *lit/lit* mice was approximately 50% of that of *lit/+* mice (*P* < 0.05, **Figure 1A**). Four-week GH injection slightly increased body mass of *lit/lit* mice (**Figures 1A**). At 13 weeks of age, mass and body mass percentage of inguinal subcutaneous fat pads were 50% and 200% greater, respectively, in *lit/lit* mice than in *lit/+* mice (*P* < 0.05, **Figures 1B, C**). However, these differences no longer existed between *lit/lit* mice injected with GH and *lit/+* littermates (**Figures 1B, C**). Overall, these data indicate that GH deficiency increased whereas GH injection reduced adipose tissue mass in mice.

### GH deficiency increased adipocyte size

**Figure 2:**
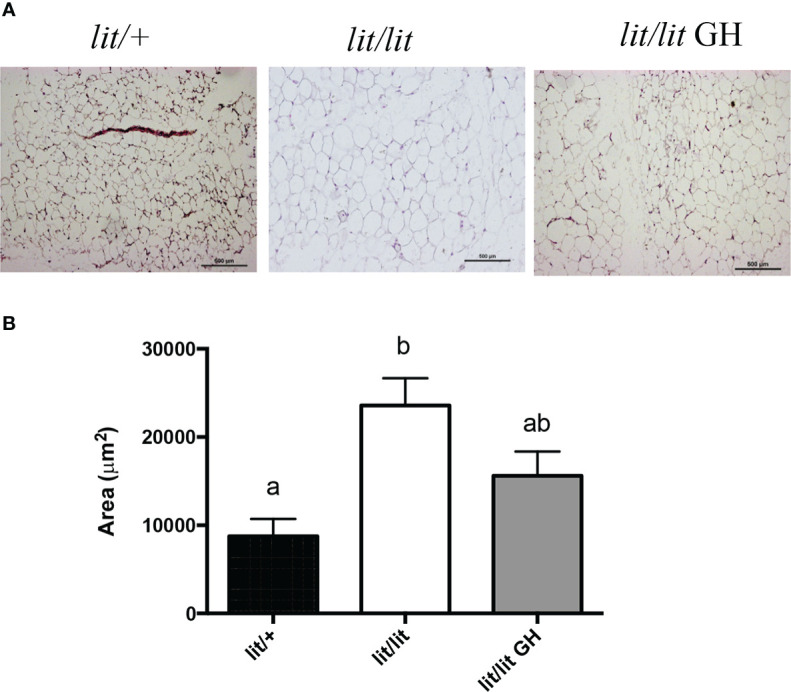
Effects of GH deficiency and GH injection on average adipocyte size in mice. Three groups of 9-week-old male *lit/lit, lit/lit*, and *lit/+* mice from the same litters were injected with GH, vehicle control, and vehicle control, respectively, once a day, for 4 weeks. Inguinal subcutaneous fat pads were collected at the end of the experiment for sectioning and staining. **(A)** Representative micrographs of fat sections. **(B)** Average adipocyte size. Data are expressed as means ± SEM (n = 5 mice). Means not sharing the same letter label are statistically different (*P* < 0.05).

To determine if GH inhibits adipose tissue mass by inhibiting adipocyte hypertrophy, we compared the sizes of adipocytes in the inguinal fat pads from 13-week-old *lit/+* mice, *lit/lit* mice, and *lit/lit* mice injected with GH for 4 weeks (**Figures 2**). Most adipocytes in *lit/lit* mice appeared to be larger than in *lit/+* mice and GH-injected *lit/lit* mice (**Figures 2A**). The average size of adipocytes in *lit/lit* mice was more than twice that of adipocytes in *lit/+* mice (*P* < 0.05, **Figures 2B**). GH injection reduced the average size of adipocytes in *lit/lit* mice by nearly 40% (**Figures 2B**). Overall, these data indicate that GH deficiency increased the size of adipocytes whereas GH injection reduced this increase in mice.

### GH injection caused adipose tissue and adipocytes to reduce in size

**Figure 3:**
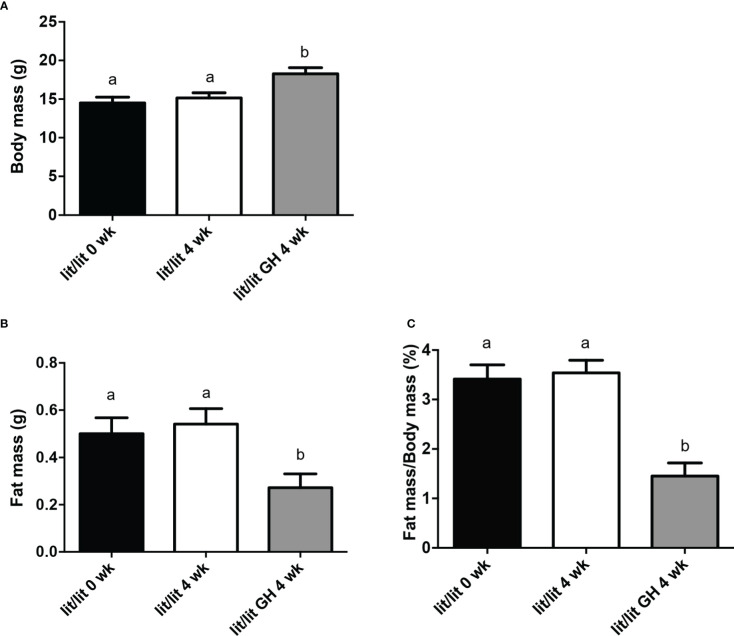
Body mass and inguinal subcutaneous fat mass of *lit/lit* mice before and after GH injection. Body mass and mass of inguinal subcutaneous fat pads were recorded from three groups of mice: 17-week-old *lit/lit* mice (indicated as *lit/lit* 0 wk in the graphs), 21-week-old *lit/lit* mice injected with GH for 4 weeks (indicated as *lit/lit* GH 4 wk in the graphs), and 21-week-old *lit/lit* mice injected with control for 4 weeks (indicated as *lit/lit* 4 wk in the graphs). **(A)** Body mass. **(B)** Fat mass. **(C)** Fat mass as percentage of body mass. Data are expressed as means ± SEM (n = 4 mice). Means labeled with different letters are statistically different (*P* < 0.05).

The results above demonstrate that GH deficiency is associated with increased adipose tissue mass and adipocyte size. We next determined if GH injection would cause mature adipose tissue and adipocytes to reduce in size. To this end, we compared the mass of inguinal fat pads and the size of adipocytes in *lit/lit* mice before (at 17 weeks of age) and after 4 weeks of injection of GH or control (at 21 weeks of age). The body mass of *lit/lit* mice without GH injection did not change significantly from 17 weeks to 21 weeks of age (**Figures 3A**). Four-week GH injection increased the average body mass of *lit/lit* mice by approximately 20% (*P* < 0.05, **Figures 3A**). However, 4-week GH injection decreased the mass and body mass percentage of inguinal fat pads in *lit/lit* mice by nearly 50% (*P* < 0.05, **Figures 3B C**).

**Figure 4:**
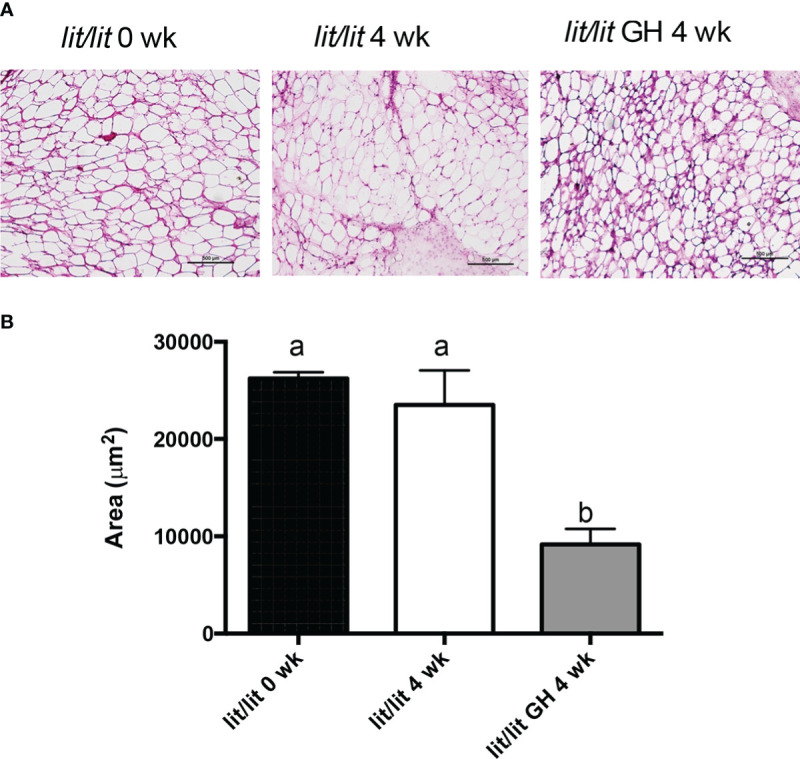
Adipocyte size of *lit/lit* mice before and after GH injection. Inguinal subcutaneous fat pads from 17-week-old *lit/lit* mice, 21-week-old *lit/lit* mice injected with GH for 4 weeks, and 21-week-old *lit/lit* mice injected with control for 4 weeks, indicated as *lit/lit* 0 wk, *lit/lit* GH 4 wk, and *lit/lit* 4 wk, respectively, in the graphs, were sectioned and stained with hematoxylin and eosin. **(A)** Representative photos of adipose tissue sections. **(B)** Average area of adipocytes. Data are expressed as mean ± SEM (n = 4 mice). Means labeled with different letters are statistically different (*P* < 0.05).

Adipose tissue from 21-week-old *lit/lit* mice injected with GH appeared to contain more small adipocytes than 21-week-old *lit/lit* mice injected with control and *lit/lit* mice before receiving GH injection (**Figures 4A**). The average size of adipocytes from 21-week-old *lit/lit* mice injected with GH for the last 4 weeks was approximately a half that from 21-week-old *lit/lit* mice injected with control or that from *lit/lit* mice before receiving GH injection (*P* < 0.05, **Figures 4B**). Overall, these data demonstrate that GH injection caused mature adipose tissue to reduce in mass and mature adipocytes to reduce in size.

### GH had no lipolytic effect on adipose explants or adipocytes from ad lib-fed mice

**Figure 5:**
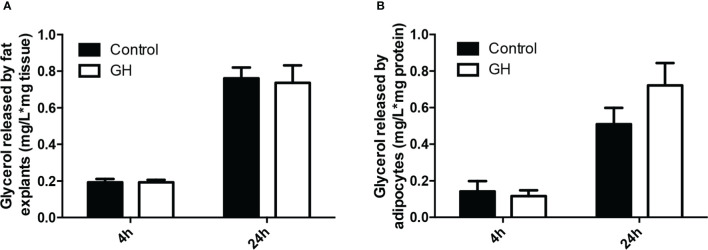
Effect of GH on lipolysis in adipose tissue and adipocytes. **(A)** Effect of GH on lipolysis in adipose tissue explants. Inguinal subcutaneous fat explants from ad lib-fed *lit/lit* mice were cultured with vehicle control or 100 ng/ml GH for 4 h and 24 h, and glycerol concentration in the medium was measured. Concentration of glycerol in the medium was normalized by the mass of adipose tissue. **(B)** Effect of GH on lipolysis in adipocytes. The stromal vascular fraction cells were isolated from ad lib-fed *lit/lit* inguinal subcutaneous fat and differentiated into adipocytes in culture. Adipocytes were then treated with control or 100 ng/ml GH for 4 h and 24 h. Glycerol concentration was normalized by total protein content of adipocytes. Data are expressed as mean ± SEM (n = 5 mice). Both 4-h and 24-h GH treatments had no effect (*P* > 0.1) on glycerol release from either adipose tissue or adipocytes. * represents multiplication.

The above results demonstrate that GH reduces adipose tissue mass and adipocyte size. One possible mechanism by which GH causes these changes is increased lipolysis. Therefore, we next determined if GH induces lipolysis in adipose tissue and adipocytes. We cultured adipose tissue explants from *lit/lit* mice with or without 100 ng/ml (~ 4.5 nM) GH for 4 h (short term) and 24 h (long term). Addition of GH to adipose explants culture did not affect glycerol release to the medium in either 4 h or 24 h of treatment (**Figures 5A**). To confirm this result, we isolated the stromal vascular fraction cells from the inguinal subcutaneous fat from *lit/lit* mice, induced the cells to form adipocytes, and then incubated the formed adipocytes with 100 ng/ml GH. Again, GH had no effect on medium concentration of glycerol in either 4 h or 24 h of GH treatment (**Figures 5B**). These data show that GH had no direct effect on lipolysis in cultured mouse adipose tissue or adipocytes.

### GH did not change isoproterenol-induced lipolysis in cultured adipose tissue or adipocytes from ad lib-fed mice

**Figure 6:**
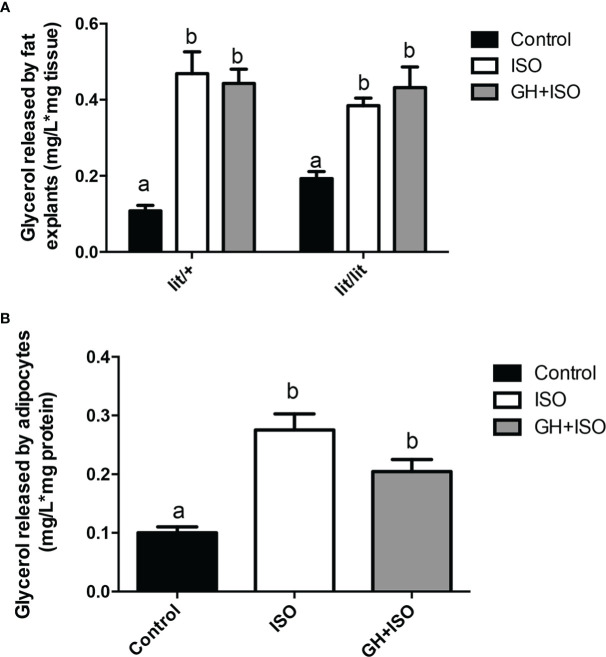
Effect of GH on isoproterenol-induced lipolysis in adipose tissue and adipocytes. **(A)** Effect of GH on isoproterenol-induced lipolysis in adipose tissue explants. Inguinal subcutaneous fat explants from ad lib-fed *lit/+* mice and *lit/lit* littermates were cultured with 10 μM isoproterenol (ISO), 10 μM ISO and 100 ng/ml GH, or vehicle control for 4 h, and glycerol released into the medium was measured. **(B)** Effect of GH on isoproterenol-induced lipolysis in adipocytes. Adipocytes were differentiated from the stromal vascular fraction cells isolated from ad lib-fed *lit/lit* inguinal subcutaneous fat. Adipocytes were cultured with 10 μM ISO, 10 μM ISO and 100 ng/ml GH, or vehicle control for 4 h, and glycerol released into the medium was measured. Data are expressed as mean ± SEM (n = 5 mice). Means labeled with different letters are statistically different (*P* < 0.05). * represents multiplication.

Multiple studies have shown that GH can enhance epinephrine-induced lipolysis (17, 27, 28). To determine if this is how GH reduces the mass of adipose tissue or the size of adipocytes in mice, we cultured the adipose tissue explants from ad lib-fed *lit/+* and *lit/lit* mice with isoproterenol, a potent beta-adrenergic agonist (29, 30), or isoproterenol in combination with GH. Isoproterenol alone stimulated a remarkable release of glycerol from both *lit/+* and *lit/lit* adipose tissue explants to culture medium (*P* < 0.05, **Figures 6A**), but the amount of glycerol released was not different between *lit/+* and *lit/lit* adipose tissue (**Figures 6A**). Treating the adipose explants from either *lit/+* or *lit/lit* mice with GH did not change the isoproterenol-induced release of glycerol (**Figures 6A**).

We also determined if GH would enhance isoproterenol-induced lipolysis in adipocytes. As indicated by the amount of glycerol released to the medium, isoproterenol had a strong effect on lipolysis in adipocytes (*P* < 0.05, **Figures 6B**), but this effect was not affected by the presence of GH (**Figures 6B**).

Overall, these data indicate that GH had no effect on isoproterenol-induced lipolysis in adipose tissue or adipocytes from ad lib-fed mice.

### GH deficiency did not affect isoproterenol-induced lipolysis in ad lib-fed mice

**Figure 7:**
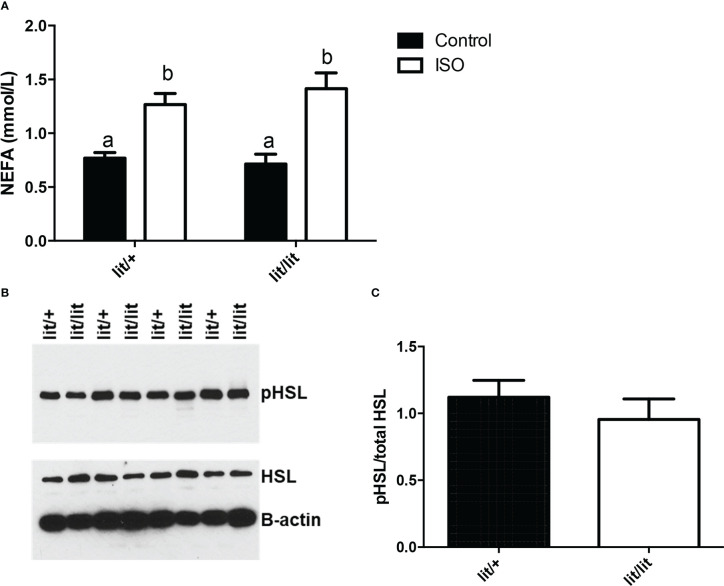
Effect of GH deficiency on isoproterenol-induced lipolysis in mice. Ad lib-fed *Lit/+* mice and *lit/lit* littermates were intraperitoneally injected with 10 μg/g body mass of isoproterenol (ISO). Blood samples were collected immediately before and 15 min after the injection, and inguinal subcutaneous fat pads were taken 15 min after the injection. **(A)** Serum concentrations of non-esterified fatty acids (NEFA). Means labeled with different letters are statistically different (*P* < 0.05, n = 4 mice). **(B)** Western blots of phosphorylated hormone-sensitive lipase (pHSL) and total HSL in adipose tissue. Beta-actin was measured as a loading control. **(C)** Density ratio of pHSL to total HSL. pHSL/HSL is not different between *lit/+* and *lit/lit* mice.

In the final experiment of this study, we compared isoproterenol-induced lipolysis in ad lib-fed *lit/+* and *lit/lit* mice. In this experiment, we injected *lit/+* and *lit/lit* mice with isoproterenol and collected blood samples immediately before and 15 min after the injection. Isoproterenol injection caused a significant increase in blood concentration of non-esterified free fatty acids (NEFA), an indication of increased lipolysis, in both *lit/+* and *lit/lit* mice (*P* < 0.05, **Figures 7A**). However, the increase was not different between *lit/+* and *lit/lit* mice (**Figures 7A**). Hormone sensitive lipase (HSL) is a key enzyme for epinephrine- or norepinephrine-induced lipolysis, and its activity is enhanced by phosphorylation (31). We compared the level of isoproterenol-induced phosphorylation in HSL in *lit/lit* and *lit/+* adipose tissue. As revealed by a Western blot analysis (**Figures 7B**), there was no difference in the abundance of phosphorylated HSL between *lit/+* and *lit/lit* adipose tissue (**Figures 7C**). These data together demonstrate that GH deficiency did not alter epinephrine- or norepinephrine-induced lipolysis in mice.

## Discussion

Although GH is essential for whole body growth, it inhibits adipose tissue growth (1–3). We conducted the present study in GH-deficient *lit/lit* mice to understand the mechanism by which GH inhibits adipose tissue mass. We found that although *lit/lit* mice weighed only a half of normal mice at the same age, the former contained significantly more subcutaneous fat, and that GH replacement treatment efficiently decreased the deposition of subcutaneous fat in *lit/lit* mice. These results are consistent with those from studying the GH receptor knockout mice (32, 33). In the present study, we also found that the epididymal fat mass was not different between *lit/+* and *lit/lit* mice (data not shown). Li and colleagues have noticed that GH receptor disruption did not affect the epididymal fat mass in mice either (34). Therefore, the subcutaneous fat appears to be more sensitive to GH than the epididymal fat, which is a major visceral fat pad in rodents (35).

Since increases in adipose tissue mass can be the result of increased adipocyte size, we determined the effects of GH deficiency and GH replacement on the size of adipocytes in mice. We found that GH-deficient *lit/lit* mice had much larger adipocytes in subcutaneous fat than *lit/+* controls. Similar result was also found in GH receptor null mice (32). These results support the mechanism that GH inhibits adipose tissue mass at least in part by inhibiting adipocyte hypertrophy. In this study, we also found that GH administration to *lit/lit* mice caused mature adipocytes to reduce in size. In line with this result, GH treatment decreased adipocyte volume in GH-deficient humans (36, 37). These results support the notion that the inhibitory effect of GH on adipocyte hypertrophy *in vivo* is at least in part mediated through increased lipolysis (13, 38).

Despite the fact that the lipolytic effect of GH *in vivo* was observed more than a half century ago (39), the underlying cellular and molecular mechanism is not completely understood. One of the questions regarding this mechanism is if GH stimulates lipolysis by directly acting on adipocytes and activating the signaling pathways leading to the activation of key lipolytic enzymes such as adipose triglyceride lipase and hormone-sensitive lipase (31). As discussed in the Introduction section of this paper, previous studies have generated seemingly conflicting answers to this question. In this study, we treated adipose tissue explants and adipocytes from *lit/lit* mice with GH or isoproterenol or both, and found that whereas isoproterenol caused marked release of glycerol from both adipose tissue explants and adipocytes, GH had no effect on glycerol release from either adipose tissue explants or adipocytes. These *in vitro* data indicated that unlike isoproterenol, GH had no direct effect on the lipolysis pathway in adipose tissue or adipocytes. Although we did not directly demonstrate GH responsiveness in the adipose tissue explants and adipocytes, which is a limitation of this study, our observation that GH had no direct lipolytic effect on adipose tissue or adipocytes from *lit/lit* mice is consistent with earlier observations that GH had no lipolytic effect in differentiated 3T3-L1 adipocytes (16), and that GH alone did not cause lipolysis in human fat explants or isolated adipocytes (17, 18). However, there have been studies showing that GH directly stimulated lipolysis (15, 40–43), perhaps through the GH receptor-mediated JAK2-STAT5 pathway (44, 45) or the MEK-ERK pathway (8). Adding to these conflicting results is a recent study finding that adipocyte-specific STAT5 knockout did not affect lipolysis in subcutaneous or inguinal adipose tissue in mice (46). These discrepancies are difficult to reconcile. Rate of lipolysis and the underlying pathway varies with the nutritional status of the animal (31, 47). Fasting is known to enhance the lipolytic effect of GH (48, 49). So, one possible cause for these seemingly conflicting results is that animals, adipose tissue, or adipocytes used in these studies differed in the nutritional status.

Growth hormone could stimulate lipolysis in adipocytes by enhancing the responsiveness of adipocytes to hormones such as epinephrine and norepinephrine, which have direct, strong effects on the lipolysis pathway (31). This possibility is supported by previous studies showing that GH increased epinephrine-induced lipolysis in cultured human adipose tissue (40), and that GH increased the function of β1 and β3 adrenergic receptors in isolated rat adipocytes (28). However, this possibility is not supported by the results from the present study. In this study, we found no difference in isoproterenol-induced lipolysis between cultured *lit/+* and *lit/lit* adipose tissue or adipocytes. We also showed that isoproterenol-induced lipolysis was not different between *lit/+* and *lit/lit* mice. Hormone-sensitive lipase (HSL) is one of the key enzymes catalyzing the hydrolysis of triglyceride (50), and is activated by epinephrine and norepinephrine upon binding to their β-receptors (51). Our data showed no difference in isoproterenol-induced phosphorylation of HSL in subcutaneous fat between *lit/+* and *lit/lit* mice. These results together argue against the notion that GH stimulates lipolysis in mice by enhancing the responsiveness of adipocytes to epinephrine or norepinephrine.

In summary, the results of the present study indicate that GH inhibits subcutaneous adipose tissue growth in mice at least in part by increasing lipolysis. However, the results of this study do not support the notion that GH stimulates lipolysis in mice by directly activating the lipolysis pathway or by enhancing epinephrine- or norepinephrine-induced lipolysis in adipocytes. Besides epinephrine and norepinephrine, hormones such as glucagon and glucocorticoids can directly induce lipolysis (31, 52). Therefore, GH might stimulate lipolysis by enhancing the responsiveness of adipose tissue to glucagon or glucocorticoids. Insulin has not only potent lipogenic but also strong antilipolytic effects (31). Long-term GH action inhibits insulin sensitivity and induces insulin resistance (53–55). Therefore, GH might stimulate lipolysis by suppressing the antilipolytic effect of insulin. Leptin is another major hormone affecting lipolysis, and interestingly, hypoleptinemia stimulates lipolysis by activating the hypothalamic-pituitary-adrenal axis (47). This suggests that GH could also affect lipolysis through a pathway commencing outside of the adipose tissue. We also cannot rule out the possibility that an unknown developmental defect caused by the *lit* mutation or an unknown mutation associated with the *lit* mutation prevents the adipose tissue of *lit/lit* mice from directly responding to GH. These and other possible indirect mechanisms by which GH stimulates lipolysis in animals remain to be studied in the future.

## Data availability statement

The original contributions presented in the study are included in the article/supplementary material. Further inquiries can be directed to the corresponding author.

## Ethics statement

The animal study was reviewed and approved by Virginia Tech Institutional Animal Care and Use Committee.

## Author contributions

LZ and HJ designed the experiments; LZ conducted the experiments; LZ analyzed the data; LZ and HL wrote the manuscript. Both authors contributed to the article and approved the submitted version.

## References

[1] Etherton TD Louveau I Sorensen MT Chaudhuri S. Mechanisms by which somatotropin decreases adipose tissue growth. Am J Clin Nutr (1993) 58(2 Suppl):287S–95S. doi: 10.1093/ajcn/58.2.287S 8328402

[2] Rosen T Bosaeus I Tolli J Lindstedt G Bengtsson BA. Increased body fat mass and decreased extracellular fluid volume in adults with growth hormone deficiency. Clin Endocrinol (Oxf) (1993) 38(1):63–71. doi: 10.1111/j.1365-2265.1993.tb00974.x 8435887

[3] Berryman DE List EO Palmer AJ Chung MY Wright-Piekarski J Lubbers E. Two-year body composition analyses of long-lived ghr null mice. J Gerontol A Biol Sci Med Sci (2010) 65(1):31–40. doi: 10.1093/gerona/glp175 19901018 PMC2796884

[4] Bengtsson BA Eden S Lonn L Kvist H Stokland A Lindstedt G. Treatment of adults with growth hormone (Gh) deficiency with recombinant human gh. J Clin Endocrinol Metab (1993) 76(2):309–17. doi: 10.1210/jcem.76.2.8432773 8432773

[5] Maison P Griffin S Nicoue-Beglah M Haddad N Balkau B Chanson P. Impact of growth hormone (Gh) treatment on cardiovascular risk factors in gh-deficient adults: A metaanalysis of blinded, randomized, placebo-controlled trials. J Clin Endocrinol Metab (2004) 89(5):2192–9. doi: 10.1210/jc.2003-030840 15126541

[6] Richelsen B Pedersen SB Borglum JD Moller-Pedersen T Jorgensen J Jorgensen JO. Growth hormone treatment of obese women for 5 wk: Effect on body composition and adipose tissue lpl activity. Am J Physiol (1994) 266(2 Pt 1):E211–6. doi: 10.1152/ajpendo.1994.266.2.E211 8141279

[7] Rosenbaum M Gertner JM Leibel RL. Effects of systemic growth hormone (Gh) administration on regional adipose tissue distribution and metabolism in gh-deficient children. J Clin Endocrinol Metab (1989) 69(6):1274–81. doi: 10.1210/jcem-69-6-1274 2685009

[8] Kopchick JJ Berryman DE Puri V Lee KY Jorgensen JOL. The effects of growth hormone on adipose tissue: Old observations, new mechanisms. Nat Rev Endocrinol (2020) 16(3):135–46. doi: 10.1038/s41574-019-0280-9 PMC7180987 31780780

[9] Chen W Hoo RL Konishi M Itoh N Lee PC Ye HY. Growth hormone induces hepatic production of fibroblast growth factor 21 through a mechanism dependent on lipolysis in adipocytes. J Biol Chem (2011) 286(40):34559–66. doi: 10.1074/jbc.M111.285965 PMC3186378 21849508

[10] Yin D Clarke SD Peters JL Etherton TD. Somatotropin-dependent decrease in fatty acid synthase mrna abundance in 3t3-F442a adipocytes is the result of a decrease in both gene transcription and mrna stability. Biochem J (1998) 331(Pt 3):815–20. doi: 10.1042/bj3310815 PMC1219422 9560309

[11] Gong JG Bramley T Webb R. The effect of recombinant bovine somatotropin on ovarian function in heifers: Follicular populations and peripheral hormones. Biol Reprod (1991) 45(6):941–9. doi: 10.1095/biolreprod45.6.941 1805998

[12] Moller N Jorgensen JO. Effects of growth hormone on glucose, lipid, and protein metabolism in human subjects. Endocrine Rev (2009) 30(2):152–77. doi: 10.1210/er.2008-0027 19240267

[13] Zhao JT Cowley MJ Lee P Birzniece V Kaplan W Ho KK. Identification of novel gh-regulated pathway of lipid metabolism in adipose tissue: A gene expression study in hypopituitary men. J Clin Endocrinol Metab (2011) 96(7):E1188–96. doi: 10.1210/jc.2010-2679 21565791

[14] Dietz J Schwartz J. Growth hormone alters lipolysis and hormone-sensitive lipase activity in 3t3-F442a adipocytes. Metabolism (1991) 40(8):800–6. doi: 10.1016/0026-0495(91)90006-I 1861630

[15] Yip RG Goodman HM. Growth hormone and dexamethasone stimulate lipolysis and activate adenylyl cyclase in rat adipocytes by selectively shifting gi Alpha2 to lower density membrane fractions. Endocrinology (1999) 140(3):1219–27. doi: 10.1210/endo.140.3.6580 10067847

[16] Frigeri LG Khoo JC Robel G. Absence of lipolytic activity from purified human growth hormone in cultured 3t3-L1 adipocytes. Horm Res (1983) 17(4):197–201. doi: 10.1159/000179698 6884983

[17] Marcus C Bolme P Micha-Johansson G Margery V Bronnegard M. Growth hormone increases the lipolytic sensitivity for catecholamines in adipocytes from healthy adults. Life Sci (1994) 54(18):1335–41. doi: 10.1016/0024-3205(94)00512-5 8190005

[18] Fain JN Cheema P Tichansky DS Madan AK. Stimulation of human omental adipose tissue lipolysis by growth hormone plus dexamethasone. Mol Cell Endocrinol (2008) 295(1-2):101–5. doi: 10.1016/j.mce.2008.05.014 18640775

[19] Fain JN Kovacev VP Scow RO. Effect of growth hormone and dexamethasone on lipolysis and metabolism in isolated fat cells of the rat. J Biol Chem (1965) 240(9):3522–9. doi: 10.1016/S0021-9258(18)97175-9 5835934

[20] Lafontan M Langin D. Lipolysis and lipid mobilization in human adipose tissue. Prog Lipid Res (2009) 48(5):275–97. doi: 10.1016/j.plipres.2009.05.001 19464318

[21] Beauville M Harant I Crampes F Riviere D Tauber MT Tauber JP. Effect of long-term rhgh administration in gh-deficient adults on fat cell epinephrine response. Am J Physiol (1992) 263(3 Pt 1):E467–72. doi: 10.1152/ajpendo.1992.263.3.E467 1415526

[22] Yang S Bjorntorp P Liu X Eden S. Growth hormone treatment of hypophysectomized rats increases catecholamine-induced lipolysis and the number of beta-adrenergic receptors in adipocytes: No differences in the effects of growth hormone on different fat depots. Obes Res (1996) 4(5):471–8. doi: 10.1002/j.1550-8528.1996.tb00256.x 8885212

[23] Godfrey P Rahal JO Beamer WG Copeland NG Jenkins NA Mayo KE. Ghrh receptor of little mice contains a missense mutation in the extracellular domain that disrupts receptor function. Nat Genet (1993) 4(3):227–32. doi: 10.1038/ng0793-227 8395283

[24] Flurkey K Papaconstantinou J Miller RA Harrison DE. Lifespan extension and delayed immune and collagen aging in mutant mice with defects in growth hormone production. Proc Natl Acad Sci U.S.A. (2001) 98(12):6736–41. doi: 10.1073/pnas.111158898 PMC34422 11371619

[25] Eleswarapu S Gu Z Jiang H. Growth hormone regulation of insulin-like growth factor-I gene expression may be mediated by multiple distal signal transducer and activator of transcription 5 binding sites. Endocrinology (2008) 149(5):2230–40. doi: 10.1210/en.2007-1344 PMC2329286 18276757

[26] Hausman DB Park HJ Hausman GJ. Isolation and culture of preadipocytes from rodent white adipose tissue. Methods Mol Biol (2008) 456:201–19. doi: 10.1007/978-1-59745-245-8_15 18516563

[27] Frick F Bohlooly YM Linden D Olsson B Tornell J Eden S. Long-term growth hormone excess induces marked alterations in lipoprotein metabolism in mice. Am J Physiol Endocrinol Metab (2001) 281(6):E1230–9. doi: 10.1152/ajpendo.2001.281.6.E1230 11701438

[28] Yang S Mulder H Holm C Eden S. Effects of growth hormone on the function of beta-adrenoceptor subtypes in rat adipocytes. Obes Res (2004) 12(2):330–9. doi: 10.1038/oby.2004.41 14981226

[29] Dugan CE Kennedy RT. Measurement of lipolysis products secreted by 3t3-L1 adipocytes using microfluidics. Methods enzymology (2014) 538:195–209. doi: 10.1016/B978-0-12-800280-3.00011-6 PMC4440587 24529440

[30] Lafontan M. Inhibition of epinephrine-induced lipolysis in isolated white adipocytes of aging rabbits by increased alpha-adrenergic responsiveness. J Lipid Res (1979) 20(2):208–16. doi: 10.1016/S0022-2275(20)40632-7 438661

[31] Duncan RE Ahmadian M Jaworski K Sarkadi-Nagy E Sul HS. Regulation of lipolysis in adipocytes. Annu Rev Nutr (2007) 27:79–101. doi: 10.1146/annurev.nutr.27.061406.093734 17313320 PMC2885771

[32] Berryman DE List EO Coschigano KT Behar K Kim JK Kopchick JJ. Comparing adiposity profiles in three mouse models with altered gh signaling. Growth Horm IGF Res (2004) 14(4):309–18. doi: 10.1016/j.ghir.2004.02.005S1096637404000292 15231300

[33] Flint DJ Binart N Kopchick J Kelly P. Effects of growth hormone and prolactin on adipose tissue development and function. Pituitary (2003) 6(2):97–102. doi: 10.1023/B:PITU.0000004800.57449.67 14703019

[34] Li Y Knapp JR Kopchick JJ. Enlargement of interscapular brown adipose tissue in growth hormone antagonist transgenic and in growth hormone receptor gene-disrupted dwarf mice. Exp Biol Med (Maywood) (2003) 228(2):207–15. doi: 10.1177/153537020322800212 12563029

[35] Chusyd DE Wang D Huffman DM Nagy TR. Relationships between rodent white adipose fat pads and human white adipose fat depots. Front Nutr (2016) 3:10. doi: 10.3389/fnut.2016.00010 27148535 PMC4835715

[36] Wabitsch M Heinze E. Body fat in gh-deficient children and the effect of treatment. Horm Res (1993) 40(1-3):5–9. doi: 10.1159/000183760 8300050

[37] Ukropec J Penesova A Skopkova M Pura M Vlcek M Radikova Z. Adipokine protein expression pattern in growth hormone deficiency predisposes to the increased fat cell size and the whole body metabolic derangements. J Clin Endocrinol Metab (2008) 93(6):2255–62. doi: 10.1210/jc.2007-2188 18334583

[38] Snyder DK Clemmons DR Underwood LE. Treatment of obese, diet-restricted subjects with growth hormone for 11 weeks: Effects on anabolism, lipolysis, and body composition. J Clin Endocrinol Metab (1988) 67(1):54–61. doi: 10.1210/jcem-67-1-54 3379136

[39] Raben MS. Growth hormone. 1. physiologic aspects. N Engl J Med (1962) 266:31–5. doi: 10.1056/NEJM196201042660109 14038540

[40] Ottosson M Lonnroth P Bjorntorp P Eden S. Effects of cortisol and growth hormone on lipolysis in human adipose tissue. J Clin Endocrinol Metab (2000) 85(2):799–803. doi: 10.1210/jc.85.2.799 10690893

[41] List EO Berryman DE Funk K Gosney ES Jara A Kelder B. The role of gh in adipose tissue: Lessons from adipose-specific gh receptor gene-disrupted mice. Mol Endocrinol (2013) 27(3):524–35. doi: 10.1210/me.2012-1330 PMC3589669 23349524

[42] Sharma R Luong Q Sharma VM Harberson M Harper B Colborn A. Growth hormone controls lipolysis by regulation of Fsp27 expression. J Endocrinol (2018) 239(3):289–301. doi: 10.1530/JOE-18-0282 30400015 PMC6226059

[43] Sharma VM Vestergaard ET Jessen N Kolind-Thomsen P Nellemann B Nielsen TS. Growth hormone acts along the ppargamma-Fsp27 axis to stimulate lipolysis in human adipocytes. Am J Physiol Endocrinol Metab (2019) 316(1):E34–42. doi: 10.1152/ajpendo.00129.2018 PMC6417689 30325658

[44] Nordstrom SM Tran JL Sos BC Wagner KU Weiss EJ. Disruption of Jak2 in adipocytes impairs lipolysis and improves fatty liver in mice with elevated gh. Mol Endocrinol (2013) 27(8):1333–42. doi: 10.1210/me.2013-1110 PMC4188962 23782652

[45] Asada N Takahashi Y Wada M Naito N Uchida H Ikeda M. Gh induced lipolysis stimulation in 3t3-L1 adipocytes stably expressing hghr: Analysis on signaling pathway and activity of 20k hgh. Mol Cell Endocrinol (2000) 162(1-2):121–9. doi: 10.1016/S0303-7207(00)00202-1 10854705

[46] Richard AJ Hang H Allerton TD Zhao P Mendoza T Ghosh S. Loss of adipocyte Stat5 confers increased depot-specific adiposity in Male and female mice that is not associated with altered adipose tissue lipolysis. Front Endocrinol (Lausanne) (2022) 13:812802. doi: 10.3389/fendo.2022.812802 35464049 PMC9022209

[47] Perry RJ Wang Y Cline GW Rabin-Court A Song JD Dufour S. Leptin mediates a glucose-fatty acid cycle to maintain glucose homeostasis in starvation. Cell (2018) 172(1-2):234–48 e17. doi: 10.1016/j.cell.2017.12.001 29307489 PMC5766366

[48] Moller L Dalman L Norrelund H Billestrup N Frystyk J Moller N. Impact of fasting on growth hormone signaling and action in muscle and fat. J Clin Endocrinol Metab (2009) 94(3):965–72. doi: 10.1210/jc.2008-1385 19066303

[49] Moller N Porksen N Ovesen P Alberti KG. Evidence for increased sensitivity of fuel mobilization to growth hormone during short-term fasting in humans. Horm Metab Res (1993) 25(3):175–9. doi: 10.1055/s-2007-1002071 8477956

[50] Yeaman SJ. Hormone-sensitive lipase–a multipurpose enzyme in lipid metabolism. Biochim Biophys Acta (1990) 1052(1):128–32. doi: 10.1016/0167-4889(90)90067-N 2182129

[51] Holm C. Molecular mechanisms regulating hormone-sensitive lipase and lipolysis. Biochem Soc Trans (2003) 31(Pt 6):1120–4. doi: 10.1042/bst0311120 14641008

[52] Xu C He J Jiang H Zu L Zhai W Pu S. Direct effect of glucocorticoids on lipolysis in adipocytes. Mol Endocrinol (2009) 23(8):1161–70. doi: 10.1210/me.2008-0464 PMC5419195 19443609

[53] Takano A Haruta T Iwata M Usui I Uno T Kawahara J. Growth hormone induces cellular insulin resistance by uncoupling phosphatidylinositol 3-kinase and its downstream signals in 3t3-L1 adipocytes. Diabetes (2001) 50(8):1891–900. doi: 10.2337/diabetes.50.8.1891 11473053

[54] del Rincon JP Iida K Gaylinn BD McCurdy CE Leitner JW Barbour LA. Growth hormone regulation of P85alpha expression and phosphoinositide 3-kinase activity in adipose tissue: Mechanism for growth hormone-mediated insulin resistance. Diabetes (2007) 56(6):1638–46. doi: 10.2337/db06-0299 17363744

[55] Dominici FP Argentino DP Munoz MC Miquet JG Sotelo AI Turyn D. Influence of the crosstalk between growth hormone and insulin signalling on the modulation of insulin sensitivity. Growth Horm IGF Res (2005) 15(5):324–36. doi: 10.1016/j.ghir.2005.07.001 16112592

